# Book Review: Tinnitus Retraining Therapy

**DOI:** 10.3389/fnins.2018.00853

**Published:** 2018-11-21

**Authors:** Amrita Pal, Daniele Ortu

**Affiliations:** Neurobehavioral Laboratory, Department of Behavior Analysis, University of North Texas, Denton, TX, United States

**Keywords:** Tinnitus Retraining Therapy (TRT), deafferentation, habituation, masking, negative reinforcement

The present review aims at integrating the classic (Jastreboff and Hazell, 2004) with more recent analyses implicating the interaction of cortical areas with limbic and paralimbic structures involved in gating processes. Jastreboff and Hazel propose a neurophysiological model and a therapeutic package for alleviating symptoms of phantom tinnitus perception following insult to the auditory pathway Tinnitus Retraining Therapy. The Tinnitus Retraining Therapy (TRT) package consists first of counseling—retraining the patient to describe tinnitus as a neutral, not aversive, stimulus. Counseling is followed by presentation of environmental natural sounds matching the tinnitus frequency so that the patient's sensory/perceptual auditory and pseudo-pain responses habituate, and are eventually considered less noxious. Within an operant conditioning paradigm, habituation is a reduction in responding due to repeated presentation of a stimulus (Catania, 1992). In the authors' model (Jastreboff and Jastreboff, 2000) attenuation of negatively valenced pseudo-pain responses coincides with the habituation of responses at the limbic and autonomic nervous system levels. In addition, according to the authors, habituation of perceptual responses may occur at a higher cortical level.

One of the prevailing etiological hypotheses describes acoustic insult causing peripheral hair cell loss or lesion in the inner ear as a prerequisite for Tinnitus, together with the interaction with cortical remapping and gating processes involving limbic and paralimbic areas, particularly the thalamus and the subcallosal area (e.g., Weisz et al., 2006; Rauschecker et al., 2010). This damage is followed by hyperactivity in the central auditory pathways due to over-representation of lesion-edge frequencies^1^ represented tonotopically in the auditory cortex. Structural reorganization associated with tinnitus is based on neuroplasticity (Møller, 2007), and appears to involve thalamic inhibitory neurons involved with sensory gating and para-limbic structures involved with emotional distress; importantly, both dopaminergic and serotonergic neuromodulatory systems appear to be involved in gating processes (Rauschecker et al., 2010, 2015). As a whole, the neuronal reorganization caused by the above described deafferentation^2^, results in the phantom pseudo-pain perception of tinnitus. More broadly, deafferentation was investigated using Transcranial Magnetic Stimulation (TMS) and functional Magnetic Resonance Imaging (fMRI) identifying a cross talk between motor, somato-sensory and the pain matrix involving the anterior cingulate cortex, parietal operculum, and the Thalamus (Hanakawa, 2012). Interestingly, auditory deafferentation does not always impact the sensory cochlear units. Therefore, the tinnitus response resulting from it cannot always be discovered behaviorally via techniques like audiometry (Weisz et al., 2006). Tinnitus may in fact involve neuronal damage in the afferent auditory pathway (and at the sub-cortical level), in the absence of cochlear damage. This brings us to an important point in the book, related to the masking procedure. The book authors prefer to call this procedure suppression instead of masking, since, a sound marginally louder than the tinnitus intensity *irrespective* of the spectrum of frequency of the masking sound, is used to mask the tinnitus perceptual response (Feldmann, 1971; Vernon et al., 1990; Jastreboff and Hazell, 2004). This procedure is also called residual inhibition, where the (residual) masking effects can last temporarily even when the masking sound is removed (Vernon et al., 1990; Roberts, 2007).

Relatedly, Roberts (2007) posits that when using a critical bandwidth frequency in tinnitus patients, the masking sound intensity needs to be only *marginally* and not extensively higher than the tinnitus intensity to generate a suppression effect. More specifically, the frequencies in the tinnitus spectrum appear to be the most efficient in masking. Roberts (2007) proposed that the selection of a critical bandwidth of frequency might involve a feed-forward inhibition of abnormal synchronous neural activity associated with the tinnitus perception at a higher level in the auditory pathway.

According to the book authors, masking can be considered from a behavioral perspective as a form of avoidance behavior. Since masking was interpreted as avoidance based, the authors mention that masking does not address the problem of tinnitus directly. Masking just postpones the tinnitus perceptual response without addressing the root cause at the peripheral sensory level. Conversely, the authors propose that by relying on the habituation procedure in the TRT package, the problem of tinnitus can be addressed more directly both at the sensory and perceptual level, without just postponing the tinnitus perceptual response as in masking. Thus, the authors note that masking, unlike TRT, could not provide long-term but just short-term relief.

Although the authors refer to the masking procedure as avoidance based, we propose here that depending on the way masking works in individual patients, the “relief” may be an outcome of either avoidance *or* escape, based on the concurrent instantiation of competing stimulus control. Technically, Catania (1992) defines avoidance and escape as negative reinforcement subcategories in which responding removes or postpones the aversive stimulus. In relation to tinnitus and masking, the patient can either escape the aversive tinnitus stimulation by generating supplementary stimulation and attending to the masking sound rather than to the ongoing unpleasant tinnitus sound. Or, in the case of avoidance, patients can generate the masking sound to pre-emptively avoid the possibility of tinnitus stimulation.

## Author contributions

All authors listed have made a substantial, direct and intellectual contribution to the work, and approved it for publication.

### Conflict of interest statement

The authors declare that the research was conducted in the absence of any commercial or financial relationships that could be construed as a potential conflict of interest.

## Footnotes

^1^Lesion-edge represents the band of frequencies encoded by the neurons just outside the damaged zone (Weisz et al., 2006).

^2^Deafferentation is the loss of afferent inputs to the brain (Hanakawa, 2012).
